# Case report: A case of anti-laminin 332 mucous membrane pemphigoid associated with severe pharyngolaryngeal involvement

**DOI:** 10.3389/fmed.2022.1000954

**Published:** 2022-09-15

**Authors:** Eleonora Quattri, Martina Zussino, Wanda Lauro, Emilio Berti, Angelo Valerio Marzano, Giovanni Genovese

**Affiliations:** ^1^Department of Pathophysiology and Transplantation, Università degli Studi di Milano, Milan, Italy; ^2^Dermatology Unit, Fondazione IRCCS Ca' Granda Ospedale Maggiore Policlinico, Milan, Italy; ^3^Dermatology and Venereology Section, University of Naples Federico II, Naples, Italy

**Keywords:** laminin 332, mucous membrane pemphigoid, pharyngolaryngeal involvement, paraneoplastic, epiligrin, laminin 5

## Abstract

A 74-year-old woman presented with a 30-day history of blisters and erosions in the oral cavity, trunk, and left eye conjunctival mucosa, also reporting a weight loss of 15 kg in the last 3 months. Histopathological examination showed subepidermal blisters and lymphocytic infiltrates with rare eosinophils in the superficial dermis. Direct immunofluorescence showed linear deposits of IgG and C3 along the dermal-epidermal junction and salt-split skin indirect immunofluorescence confirmed the presence of linear deposits of IgG along the blister floor. Indirect immunofluorescence revealed antibodies against laminin 332 using recombinant laminin 332 expressed in human HEK293 cells, and commercial ELISA kits (Euroimmun, Padova, Italy) did not reveal antibodies against BP230 and BP180 antigens. Anti-laminin 332 mucous membrane pemphigoid (MMP), a condition often associated with a hidden neoplasm, was diagnosed. In our case, the paraneoplastic nature of MMP was excluded. Thus, topical treatment with triamcinolone acetonide 0.1% in orabase once daily for 30 days, oral prednisone 0.75 mg/kg/day and rituximab were started to relieve symptoms. Conjunctival, nasal and oral erosions improved, as well as skin lesions, but later the patient was tracheotomized due to respiratory distress linked to the appearance of pharyngolaryngeal synechiae.

## Introduction

Mucous membrane pemphigoid (MMP) encompasses a heterogeneous group of subepithelial autoimmune blistering diseases mediated by autoantibodies against different adhesion molecules of the epithelial basement membrane zone (BMZ) such as BP180, BP230, collagen VII, integrin α6β4 and laminin 332 (1, 2). It predominantly affects the mucous membranes and is characterized by linear deposition of IgG, IgA or C3 along the epithelial basement membrane (1, 2). The diagnosis is confirmed by histology and direct and indirect immunofluorescence techniques; more expensive techniques such as laminin-332 transfected cell technique are more rarely performed (3). In MMP patients with anti-laminin 332 autoantibodies, an increased risk for neoplasms associated with a poor prognosis – especially within the 1st year from MMP onset – has been described (4–6). Egan et al. (4) reported in these patients a relative risk for cancer of 6.8, similar to that of adults with dermatomyositis. Consistent with these data, some authors regarded anti-laminin 332 MMP as a paraneoplastic syndrome triggered by an autoimmune response to laminin 332 expressed in the tumor cells and in the normal mucosal tissue. (7) Furthermore, some case reports pointed out a clinical remission of anti-laminin 332 MMP after treatment of the associated neoplasm (8–10). More recently, however, three independent studies did not confirm the association between anti-laminin 332 autoantibodies and cancer in their cohort of patients (11–13). Significant correlations between serum levels of anti-laminin 332 antibodies and disease activity have been shown by means of ELISA, (14) immunoblotting and IIF (6) and in a recent meta-analysis of 200 published MMP cases an association of anti-laminin 332 antibodies with pharyngo-laryngeal and oro-pharyngo-laryngeal (15) involvements has been found.

Herein, we describe a case of anti-laminin 332 MMP with severe pharyngolaryngeal involvement which was not associated with neoplasms.

## Case description

**Figure 1:**
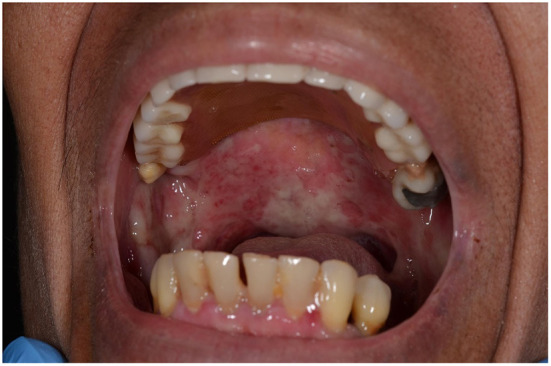
Blisters and erosions in the oral cavity of our patient.

**Figure 2:**
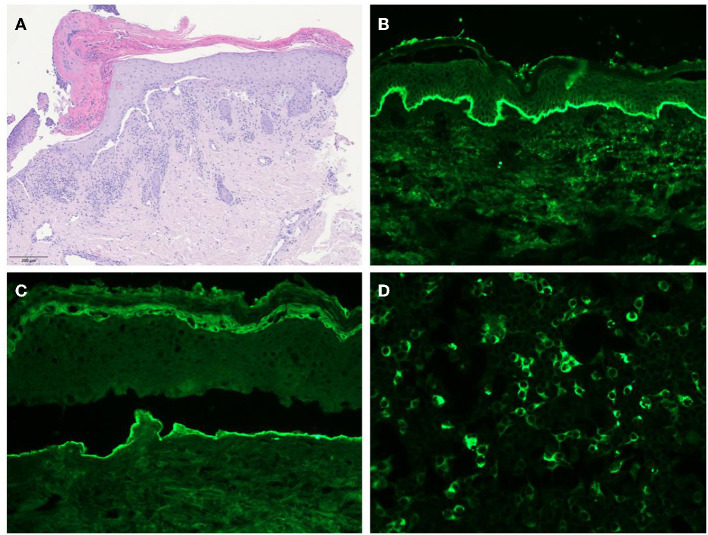
**(A)** Histology showing subepidermal blisters and lymphocytic infiltrates with rare eosinophils in the superficial dermis. **(B)** Direct immunofluorescence revealing IgG deposits along the dermal-epithelial junction. **(C)** Salt-split skin indirect immunofluorescence showing IgG deposits along the floor of the blister. **(D)** Indirect immunofluorescence test using recombinant laminin 332 expressed in human HEK293 cells revealing the presence of anti-laminin 332 antibodies in the patient's serum.

A 74-year-old woman presented with a 30-day history of blisters and erosions in the oral cavity, trunk, and left eye conjunctival mucosa. Even if her medical history was unremarkable, the patient reported a weight loss of 15 kg in the last 3 months. She denied recent intake of new drugs. Dermatological examination revealed erosions in the aforementioned areas and in the nasal mucosa (Figure 1). Eye examination confirmed synechiae in both eyes, while the otolaryngological evaluation excluded the presence of pharyngolaryngeal synechiae. Differential diagnoses included pemphigus *versus* MMP. Histopathological examination showed subepidermal blisters and lymphocytic infiltrates with rare eosinophils in the superficial dermis (Figure 2A). Direct immunofluorescence showed linear deposits of IgG and C3 along the dermal-epidermal junction, while indirect immunofluorescence confirmed the presence of linear deposition of IgG along the basement membrane (Figure 2B). Commercial ELISA kits (Euroimmun, Padova, Italy) did not reveal antibodies against BP230 and BP180 antigens. Indirect immunofluorescence on salt split skin confirmed the linear IgG deposition along the blister floor (Figure 2C). We demonstrated the presence of anti-laminin 332 antibodies in the patient's serum using a sensitive and highly specific indirect IF test using recombinant laminin 332 expressed in human HEK293 cells, with all laminin subunits α3, β3, and γ2 (Figure 2D), as already performed in other studies (6, 12, 16). We made a diagnosis of anti-laminin 332 MMP and requested in-depth blood chemistry and instrumental examinations due to the suspicion of a hidden neoplasm. In the meantime, the patient was given topical treatment with triamcinolone acetonide 0.1% in orabase once daily for 30 days and oral prednisone 0.75 mg/kg/day to relieve symptoms. Lymph node and abdomen ultrasound, mammography and total body computed tomography were oncologically negative. Paraneoplastic MMP was ruled out, so the patient was started on infusion therapy with rituximab. Conjunctival, nasal and oral erosions rapidly improved as well as skin lesions. Only mild itching persisted. At the time of diagnosis, Mucous Membrane Pemphigoid Disease Area Index score (MMPDAI) was 44 points, reduced to 15 during follow-up. However, 6 months after rituximab treatment the patient was tracheotomized for acute respiratory failure due to pharyngolaryngeal synechiae, a difficult-to-prevent outcome in MMP.

## Discussion

In the last decade, there has been mounting body of evidence that patients' subsets identified by the binding of their serum autoantibodies to specific target antigens may have different disease phenotypes, clinical outcomes and association with malignancy. In particular, retrospective studies have highlighted an increased risk for cancer in anti-laminin 332 MMP. Laminin 332, formerly called epiligrin or laminin 5, is a major component of the BMZ and consists of three subunits (α3, β3, and γ2). It is localized at the interface between lamina lucida and lamina densa and contributes to anchoring hemidesmosomal structures such as BP180 and integrin α6β4 to collagen VII. (17) Antibodies against laminin 332 are reported in 12 up to 75% of MMP patients (11, 12, 18–21). The pathogenicity of anti-laminin 332 antibodies has been demonstrated by the passive transfer of IgG against laminin 332 into neonatal and adult mice (21, 22). The pathogenic mechanisms explaining the differences in terms of cancer risk between patients with anti-laminin 332 MMP and anti-integrin α6β4 MMP are still a matter of speculation. Laminin 332 is expressed in the extracellular matrix of different neoplasms and its levels may be over- or under-represented based on the tumor type. (19) Laminin 332 has been proposed to act as an oncosuppressor molecule, whose increased cleavage by means of tumor-associated proteases may promote cancer progression. (20) Thus, anti-laminin 332 antibodies can be hypothesized to determine *in vivo* alterations mimicking the action of these proteases by inhibiting laminin 332 or activating tumor-associated proteases (21). However, in the light of *in vitro* inhibiting properties on tumor growth of anti-laminin 332 antibodies (11), it can also be postulated that anti-laminin 332 antibody production may be due to a paraneoplastic autoimmune response toward common molecules shared by the tumor and adhesion structures of the BMZ.

However, in contrast with the retrospective studies that have highlighted an increased risk for cancer in anti-laminin 332 MMP, more recent studies failed to confirm the association between anti-laminin 332 MMP and cancer (11–13). In our case, accurate instrumental investigations apparently rule out any hidden neoplasm. On the other hand, in line with literature data showing an association of anti-laminin 332 antibodies with severe pharyngo-laryngeal and oro-pharyngo-laryngeal involvements (15), our patient developed acute respiratory failure due to pharyngolaryngeal stenosis. In conclusion, potential complications linked to high morbidity and/or mortality, such as esophageal/laryngeal stenosis, are relatively frequent in anti-laminin 332 MMP and require a prompt diagnosis and an effective treatment in order to delay or halt the disease progression.

## Data availability statement

The original contributions presented in the study are included in the article/supplementary material, further inquiries can be directed to the corresponding author/s.

## Author contributions

EQ and GG wrote the paper. MZ and WL contributed in drafting the manuscript. EB performed laboratory tests. AM critically reviewed the manuscript. All authors contributed to the article and approved the submitted version.

## Funding

This study was partially funded by Italian Ministry of Health, Current research IRCCS.

## Conflict of interest

The authors declare that the research was conducted in the absence of any commercial or financial relationships that could be construed as a potential conflict of interest.

## Publisher's note

All claims expressed in this article are solely those of the authors and do not necessarily represent those of their affiliated organizations, or those of the publisher, the editors and the reviewers. Any product that may be evaluated in this article, or claim that may be made by its manufacturer, is not guaranteed or endorsed by the publisher.
