# Corrigendum: Case report: A case of anti-laminin 332 mucous membrane pemphigoid associated with severe pharyngolaryngeal involvement

**DOI:** 10.3389/fmed.2022.1100467

**Published:** 2022-12-15

**Authors:** Eleonora Quattri, Martina Zussino, Wanda Lauro, Emilio Berti, Angelo Valerio Marzano, Giovanni Genovese

**Affiliations:** ^1^Department of Pathophysiology and Transplantation, Università degli Studi di Milano, Milan, Italy; ^2^Dermatology Unit, Fondazione IRCCS Ca' Granda Ospedale Maggiore Policlinico, Milan, Italy; ^3^Dermatology and Venereology Section, University of Naples Federico II, Naples, Italy

**Keywords:** laminin 332, mucous membrane pemphigoid, pharyngolaryngeal involvement, paraneoplastic, epiligrin, laminin 5

In the published article, there was an error in the **Funding** statement. [There was no funding statement]. The correct **Funding** statement appears below.

